# Phylogeographic Diversity Analysis of *Bipolaris sorokiniana* (Sacc.) Shoemaker Causing Spot Blotch Disease in Wheat and Barley

**DOI:** 10.3390/genes13122206

**Published:** 2022-11-24

**Authors:** Pradeep Sharma, Shefali Mishra, Garima Singroha, Rajan Selva Kumar, Sanjay Kumar Singh, Gyanendra Pratap Singh

**Affiliations:** 1 ICAR—Indian Institute of Wheat and Barley Research, Agrasain Marg, Karnal 132001, India; 2 ICAR—Sugarcane Breeding Institute, Coimbatore 641001, India; 3 ICAR—Indian Agricultural Research Institute, New Delhi 110001, India

**Keywords:** *B. sorokiniana*, ITS, *GAPDH*, *TEF-1α*, demographic history, genetic diversity

## Abstract

*Bipolaris sorokiniana* is a fungal pathogen that infects wheat, barley, and other crops, causing spot blotch disease. The disease is most common in humid, warm, wheat-growing regions, with South Asia’s Eastern Gangetic Plains serving as a hotspot. There is very little information known about its genetic variability, demography, and divergence period. The current work is the first to study the phylogeographic patterns of *B. sorokiniana* isolates obtained from various wheat and barley-growing regions throughout the world, with the goal of elucidating the demographic history and estimating divergence times. In this study, 162 ITS sequences, 18 *GAPDH* sequences, and 74 *TEF-1α* sequences from *B. sorokiniana* obtained from the GenBank, including 21 ITS sequences produced in this study, were used to analyse the phylogeographic pattern of distribution and evolution of *B. sorokiniana* infecting wheat and barley. The degrees of differentiation among *B. sorokiniana* sequences from eighteen countries imply the presence of a broad and geographically undifferentiated global population. The study provided forty haplotypes. The H_1 haplotype was identified to be the ancestral haplotype, followed by H_29 and H_27, with H_1 occupying a central position in the median-joining network and being shared by several populations from different continents. The phylogeographic patterns of species based on multi-gene analysis, as well as the predominance of a single haplotype, suggested that human-mediated dispersal may have played a significant role in shaping this pathogen’s population. According to divergence time analysis, haplogroups began at the Plio/Pleistocene boundary.

## 1. Introduction

Spot blotch of wheat and barley caused by the fungus *Bipolaris sorokiniana* (Sacc.) Shoemaker, belonging to the order Pleosporales (*Pleosporaceae* family), is one of the major production constraints in warmer areas worldwide [1,2]. The infection begins as tiny, dark-brown scratches that progress to light- to dark-brown oval or elongated blotches. With the progression of disease, these elongated blotches join together to form necrotic lesions, leading to cell death. (*Cochliobolus sativus*) Drechs. Ex Dastur is the sexual (perfect) stage for this fungus. Except for Zambia, *C. sativus* has not been discovered in nature; however, sexual reproduction in this pathogen has been documented very rarely [3,4]. *B. sorokiniana*, on the other hand, reproduces primarily by the generation of asexual conidia [2].

Yield losses are reported to vary from 15.5 to 19.6% and may be up to 100% under congenial conditions in vulnerable genotypes, reducing the seed quality [2,5]. Spot blotch infection results in shrivelled, discoloured, and black pointed seeds, affecting grain quality and trade. The fungus has been shown to infect wild as well as cultivated Poaceae species [6]. The wide host range of *B. Sorokiniana* results in huge genetic variability of the pathogen [7,8,9]. Although fungicides can be used to reduce damage from the disease, a more economical and environmentally desirable means of control is through the deployment of resistant cultivars. Understanding the pathogen’sgenetic variability will aid in the development of long-lasting resistant wheat varieties.

The high genetic variability among fungal pathogen populations across a wide range of environments, as well as the involvement of minor genes in governing resistance in foliar blight or spot blotch breeding programs, continues to be a challenge for breeders. Considering the yield losses caused by the fungus *B. sorokiniana*, understanding its genetic diversity throughmolecular characterization is critical. The analysis of the ITS (internal transcribed spacer) region has been widely used in studying fungal phylogenetic relationships [10]. Its short length, rapid evolution rate, and availability of universal primers make the internal transcribed spacer (ITS1 and ITS2) the most extensively used molecular markers for systematic studies. Polymorphic ITS sequences are the result of hybridization, recombination, and pseudogenization of cistrons. Secondary structure stability, conserved motifs, substitution rates, and phylogenetic locations can all be used to identify ITS sequence polymorphisms caused by non-functional pseudogenes. Studies on the morphological, genetics, and molecular markers of *B. sorokiniana*, which causes spot blotch disease in wheat, have been reviewed elsewhere [2].

In the past, several molecular techniques such as cleaved amplified polymorphic sequence (CAPS) and random amplified polymorphic DNA [11] have been performed for ITS region analysis for pathogen characterization [10,12,13]. *B. sorokiniana* is a variable fungus due to somatic hybridization, sexual reproduction, and heterokaryosis [14,15]. The Eastern Gangetic Plains of South Asia area declared hotspot of leaf blotch disease [2,16]. The sexual stage of compatible isolates of *B. sorokiniana* showed a significant degree of diversity from various geographic regions [4]. A phylogeographic analysis provides a better understanding of the impact of historical events on genetic diversity patterns within and across species [17]. Zhong et al. [18] developed a rapid and reliable PCR-based diagnostic marker capable of detecting the pathogen in pre-symptomatic wheat leaves as well as soil. Even markers from *Pyrenophorateres* f. sp. *maculata* have also been utilized for evaluating variations among *B. Sorokiniana* isolates [19]. EST-SSRs and ribosomal DNA polymorphism, including sequence variation in the ITS characterization of fungal isolates, have been used by several research groups [20,21,22,23,24]. Sun et al. [25] also reported the genetic variability of Chinese isolates of *B. sorokianana* using ITS, *β-tubulin*, and *TEF-1α* gene markers [26]. Bhunjun et al. [27] also reported *Bipolaris* species delimitation using ITS and *glyceraldehyde-3-phosphate dehydrogenase* gene (*GPDH*). *Brn1* gene sequences from *B. sorokiniana* were also utilised for designing the primer pairs. Information regarding species diversity at geographical level is a prerequisite for developing disease-resistant cultivars and disease management. The aim of this study was to understand the phylogeographic patterns of *B. sorokiniana* isolates infecting wheat and barley worldwide using ITS, *GPDH*, and *TEF-1α* sequences to elucidate the demographic history and divergence time estimation. The diversity analysis will not only aid in the designing and improving of wheat-breeding programmes to develop new cultivars, but also aid in the development of effective chemical management strategies for the control of leaf blight disease.

## 2. Materials and Methods

### 2.1. Collection of Diseased Samples, Isolation of Bipolaris Isolates and Their Maintenance

Fungal-infected leaves of wheat and barley were collected from different locations in India (Supplementary Table S1). Small mycelial chunks of infected leaf tissues were surface sterilized with successive washes of 75% ethanol (25 s), 0.1% mercuric chloride solution (1.5 min), three rinses with sterile distilled water, and then left to air dry and transferred to potato dextrose agar (PDA) and incubated at 22 ± 2 °C for fungal growth. Colonies arising froma single spore were used to generate pure fungal cultures (Sharma et al., 2005, [28]). Conical flasks containing 150 mL of autoclaved potato dextrose broth were inoculated with fungal mycelia from 7-day-old cultures and were incubated at 22 ± 2 °C in an orbital incubator shaker (Thermo Scientific-MaxQ^TM^, Waltham, MA, USA) (100 rpm). After a week, fungal mycelia were harvested using double-layered filter paper and dried in between sterilized filter layers under a laminar flow cabinet. The dried mycelia were kept at −20 °C for molecular research.

### 2.2. DNA Extraction and PCR Amplification

For extraction of fungal genomic DNA, 40 mg of dried mycelium of each isolate of *B. sorokiniana* was processed using the CTAB method as described with minor modification [29]. A UV/VIS spectrophotometer (Simadzu, Kyoto, Japan) was used for DNA quantification, and the DNA was stored at −20 °C for further use. To amplify the ITS region in the rDNA gene fragments of *B. sorokiniana* isolates, fungal specific universal primers (ITS1 and ITS4) were used [30]. PCR amplification conditions were used as mentioned earlier [28]. The amplified product was separated using agarose gel electrophoresis and viewed under the αInnotech gel documentation system (Alphaimager, Houston, TX, USA). The band of amplified product was cut out from the gel and was cleared of any agarose residue using the QIA quickgel extraction kit (Qiagen, Hilden, Germany) as per the manufacturer’s instructions. The purified amplicon thus obtained was sequenced by (Eurofins Genomics Pvt Ltd., Bengaluru, India) using the ITS1 and ITS4 PCR primers. Sequences were generated from two independent sequencing reactions for each sample and were deposited to NCBI (Supplementary Table S1).

### 2.3. Phylogenetic Analysis

In the present study, 162 ITS sequences, 18 of *GAPDH* and 74 *TEF-1α* sequences of *B. sorokiniana* retrieved from NCBI, including 21 ITS sequences obtained in this study, were used to perform multiple sequence alignment (MSA) using ClustalW of MEGA-X [31] as well as to construct the phylogenetic tree using the JTT model with γ distribution and complete deletion of removal or gaps. Finally, the tree was visualized using the iTOL (iTOL v6 verson; iTOL.embl.de) (interactive tree of life) software.

### 2.4. Genetic Diversity and Haplotype Relationship

The final dataset with 254 gene sequences consisting of ITS, *TEF*-1α, and *GAPDH* was used for combined analysis (Supplementary Table S2). To interpret sequence variation and describe populations, the following parameters were calculated in DnaSP v. 5.101 [32]: the number of polymorphic sites (S), average number of nucleotide differences (k), nucleotide diversity (π), number of haplotypes (h), total number of mutations (η), and the haplotype diversity (Hd).

Using NETWORK ver. 4.6 [33], a network was constructed using the median-joining algorithm to describe the distribution and geographical relationships between the populations of *Bipolaris sorokiniana*. For geographical grouping of populations, AMOVA was performed in Arlequin 3.5 [34].

### 2.5. Demographic History and Divergence Time

To determine if *B. sorokiniana* populations have undergone any population expansion in the recent past, we constructed mismatch distributions and compared them with the predicted distributions from population expansion models [35]. The DnaSP v. 5.101 was used to determine the calculation of class I neutrality tests: Tajima’s *D* [36], and Fu’s *Fs* [37] population size change test R^2^ for detecting departure from the mutation/drift equilibrium.

The significance of the test was built on 1000 coalescent simulations. A zero value of Tajima’s *D* indicates neutrality, while a significant value is representative of heterogeneous mutation rate and population expansion. A higher negative value of Fu’ *Fs* indicates population diversity, and lower R^2^ values are predicted under this population growth expansion.

The R^2^ test performs well even with a limited sample size. The distribution of genetic variations between pairs of haplotypes (mismatch distribution) was calculated using the same program and the premise of panmictic populations. In general, multimodal distributions are compatible with demographic stability or numerous expansion events, whereas unimodal distributions commonly indicate that the population has recently expanded in both population and geographic terms [38]. The mismatch distribution was calculated using all populations combined, as well as by geographic distribution and haplotype groups.

BEAST 1.8.3 [39] was used to estimate the time since the most recent common ancestor using a Bayesian method. Analyses were carried out using ITS sequences from *Biloparis sorokinana* populations, including *Bipolaris oryzae* as an out-group, using the GTR nucleotide substitution model, which was found to be the best model for the data in MEGA7 [24]. We utilised an uncorrelated relaxed clock with a lognormal relaxed distribution. The previous coalescent tree was of constant size, which is ideal for trees that describe connections between individuals in the same population or species. The analysis was carried out using default parameters for the Markov Chain Monte Carlo (MCMC), setting 10 million generations and discarding 10% of the first runs. Tree Annotator v1.8.0 was used to merge the resultant trees, and FigTree v1.4.0 was used to display the consensus tree with the divergence periods [40].

## 3. Results

### 3.1. Analysis of Genetic Diversity and Phylogenetic Relationships

**Figure 1:**
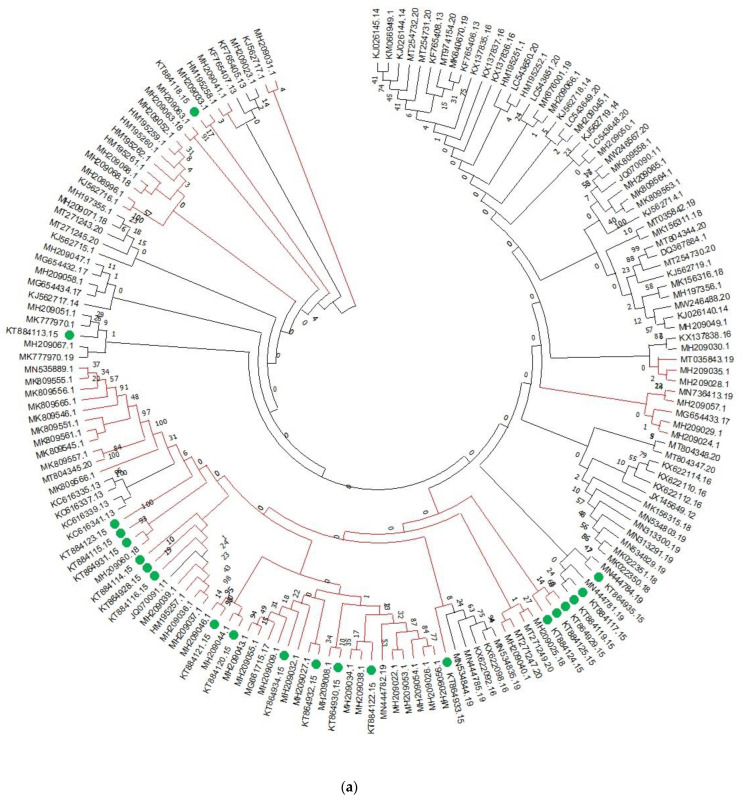

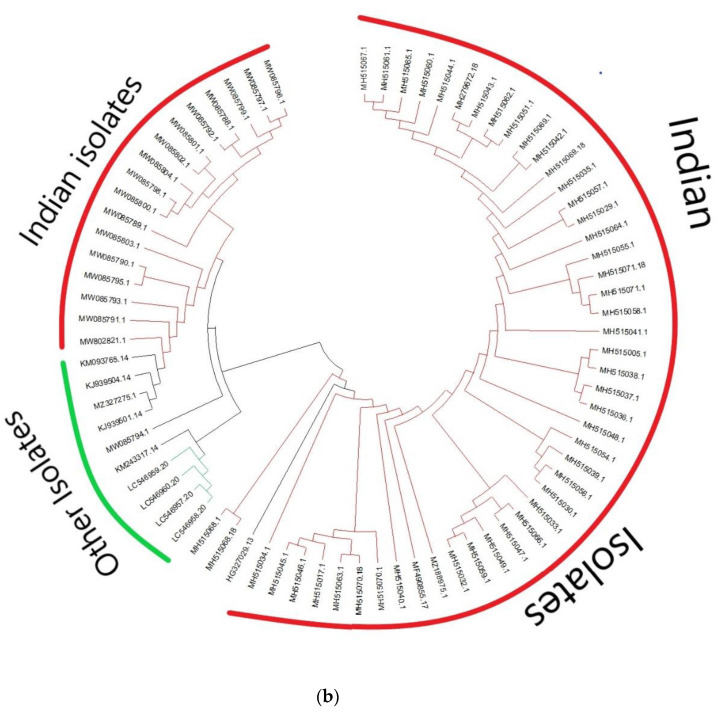

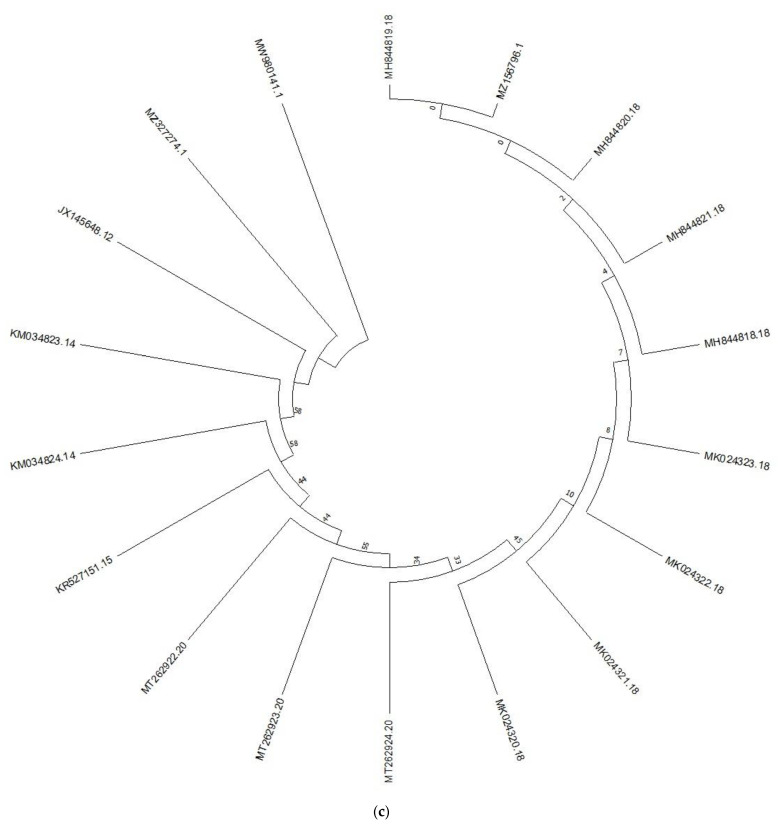
Phylogenetic analysis of (**a**) ITS, (**b**) *TEF-1α*, and (**c**) *GAPDH* nucleotide sequences of *B. sorokiniana*.

PCR amplicons of ~550 bp obtained from all the 21 isolates of *B. sorokiniana* were sequenced bidirectionally. The sequences thus obtained had been deposited in GenBank (Supplementary Table S1). The average nucleotide composition of the ITS sequences of *B. sorokiniana* represented 20.12% A, 20.13% C, 20% and 50.5% G+C content. For the analysis, we downloaded 141 ITS region sequences available in the NCBI database and phylogenetic analysis was carried out (Figure 1a). The evolutionary relationships among the *B. sorokiniana* were discovered using a phylogenetic tree, which resulted in three groupings (Figure 1a). Except for the isolates KT884114 and KT88413 in clade I, the majority of the present studied isolates were grouped in cladeII, which contains ITS sequences from different geographic regions (Figure 1a), followed by KT884124, KT884125, and KT884939 in clade III, while using 74 *TEF-1α* sequence analysis shows that the majority of the Indian isolates formed separate clusters except for the 17 isolates which clustered with isolates from other countries (Figure 1b). While using 18 *GAPDHs*, different results were obtained (Figure 1c). In contrast, the phylogenetic tree was well-supported and corresponded to the haplotype network.

A total of 40 diverse haplotypes (Hd = 0.71 ± 0.02) were identified in 254 sequences of *B. sorokiniana* (Table 1). However, nucleotide diversity (π) was 0.33 ± 0.019 across all populations, the average difference in the number of nucleotides (K) among the sequences was 75.5 (Table 1), and the average number of segregating sites was 225. Therefore, each population is a representative of the overall diversity of the species.

### 3.2. Haplotype Relationship

**Figure 2:**
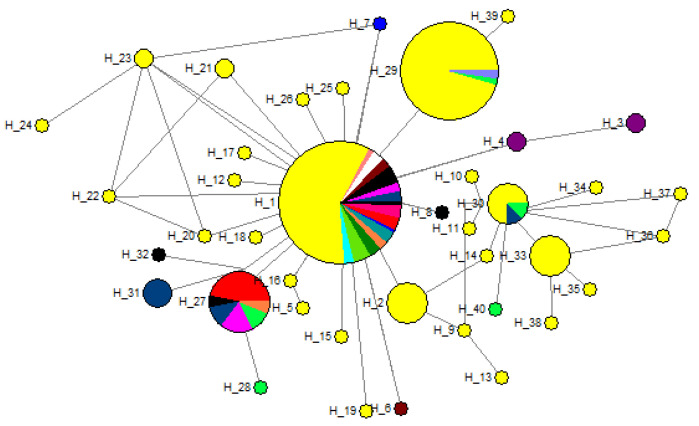
Median joining network of rDNA haplotype of *B. sorokiniana* populations. The size of the circle is related with the frequency of haplotypes and different colours indicate the proportion of individual samples in different populations within the study area.

We constructed a median joining tree utilising 254 sequences based on ITS, *GAPDH*, and *TEF-1α* genes to determine the population structure and evolutionary ancestry of *B. sorokiniana* (Figure 2). The network configuration in the *B. sorokiniana* lineages shows a reticulate network with a star-shaped divergence at the tip. Furthermore, the majority of the haplotypes were found to be linked to H_1, the most common haplotype, which consists of 127 isolates from various regions, followed by H_29, the second most common haplotype, which has 45 sequences. Other haplotypes with lower gene counts include H_27, which has 17 sequences, and H_2, H_30, and H_33, which each contain eight sequences and were linked by a single or maximum of three mutation phases. Except for a few Asian countries, haplotype H_1 was detected in the network’s core and was shared by all *B. sorokiniana* populations around the globe. The haplotype H_27, on the other hand, represents a population that is geographically varied and all had a frequency below 5%. Further investigation focused on haplogroups, which were defined geographically based on the network’s topology.

Analysis of molecular variance (AMOVA) represented a low value of *F_ST_* (0.05) when considered as a single group comprising all the populations. This analysis revealed 5.8% interspecies variation and 94.15%intraspecies variation (Table 2). This study proposes a weak genetic structural variation but underpins the action of gene flow. The F-statistic value was found to be very low (0.18) when the populations were analysed based on geographical grouping and the percentage of variation was 7.44% among groups, 27.77% among populations within groups, and 91.28% within population within groups (Table 2).

### 3.3. Population Demography Events and Divergence Time

**Figure 3:**
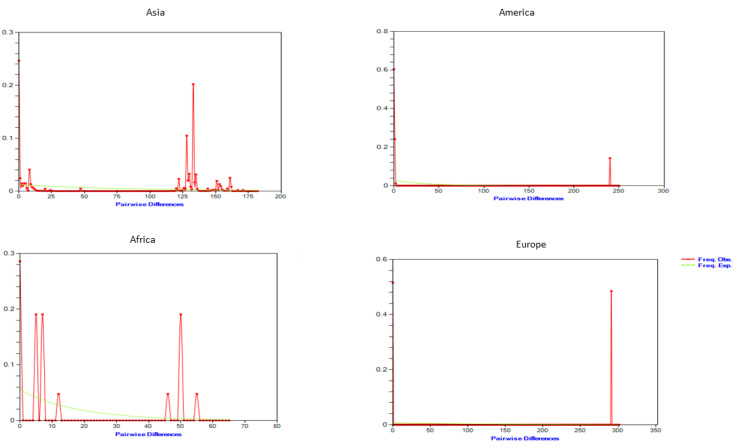
Mismatch distribution relevant to geographic and haplotypic groups of *B. sorokiniana*. The observed frequency of pairwise differences (solid line) iscompared against the expected frequencies under expanding population size (dotted line), whereas figures in the clockwise direction representmismatch distribution across the continents of Africa, America, Asia, and Europe, respectively.

By examining the frequency distribution of pairwise differences across sequences, Roger [38] proposed demographic expansions in the past. Recent demographic growth is indicated by a unimodal distribution derived from the mismatch distribution of total samples. On a single geographical continent, the multimodal pattern was identified. Haplogroups from Morocco and Egypt from the African continent (Figure 3) showed a multimodal pattern, but groups from other continents, e.g., America and Europe, showed a unimodal pattern, reflecting a recent population expansion or range extension with a high degree of movement within neighbourhoods [34].

**Figure 4:**
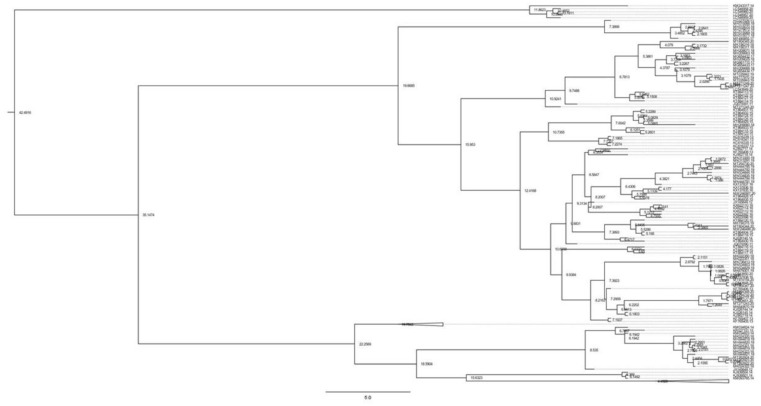
Chronogram with divergence times. Numbers annotated on each node represent the divergence times between clades and the standard deviations (95% of high posterior density) are represented by the blue bars.

The demographic history of earlier population increases was deciphered using Tajima’s *D* and Fu’s *Fs* tests. The findings of Tajima’s *D* test and Fu’s *Fs* test are shown in Table 1. For numerous groups, Tajima’s *D* values were significant, including China, Turkey, Azerbaijan, Argentina, and Kazakhstan. However, except for Syria, Fu’s *Fs* were positive, and *p*-values for numerous groups were statistically significant, showing that there was an excess of low-frequency polymorphisms relative to expectation, indicating that the null hypothesis of population neutrality could be rejected (Table 2). The low R^2^ score, on the other hand, showed recent growth expansion. *B. sorokiniana* and its outgroup (*B. oryzae*) split 2.1 million years ago (Mya). Divergence within *B. sorokiniana* began 1.2 Mya, in the middle of the Pleistocene period. The divergence of *B. sorokiniana* in Asian countries occurred 0.47 Mya. It was discovered that divergence between Indian isolates and American isolates began around 0.35 Mya. Our research focused on determining the time of divergence of Asian isolates (particularly Indian samples), and we discovered that Indian lineages diverged around 0.38 Mya. We also discovered that the Japan, Russia, Jordan, and China lineages broke off at 0.16 Mya (Figure 4). The divergence between Indian and African isolates, on the other hand, occurred about 0.05 Mya.

## 4. Discussion

Spot blotch has emerged as one of the most important pathogens, particularly with regard to wheat and barley production throughout the world, including Southeast Asia [2,21]. Host resistance is an economic and viable approach for managing the pathogen. However, these measures have not yielded satisfactory results thus far. Spot blotch variation is extremely useful for identifying resistant germplasm, deploying cultivars with different resistance genes, and analysing the emergence of new pathotypes. A genetically diverse pathogen population screening is required for an effective resistance-breeding programme. As a result, understanding the pathogen’s genetic variation is critical for developing effective management strategies. Moreover, genetic variability among naturally occurring populations is an outcome of evolution and demographic processes and provides an important tool to determine the evolutional potential of a species [41]. Here, we examined the intraspecific genetic variability of *B. sorokiniana* (using ITS as well as with genes *GAPDH* and *TEF1α* region of *B. sorokiniana*) to acknowledge the evolution and phylogeography of the species.

According to phylogenetic study, *B. Sorokiniana* was a single species with no distinct groups, since all of the sequences taken from different countries were clustered together in a single group and outgroups were put into separate groups. Haplotype diversity is influenced by mutation, marker discovery, recombination, and demography [42]. Based on three gene sequences, 40 haplotypes were identified in a group of 254 isolates, with the predominant haplotype H_1 comprising 127 individuals (50% of total isolates) with distinct geographies and host specificities. These results were indicative of high gene flow occurring among the geographically distinct populations of the pathogen. The haplotype network has a dispersed topology due to its high singleton ratio. This type of phylogenetic inference suggests a recent population expansion from a small number of founders following a genetic bottleneck [41]. The results of median-joining network analysis of *B. Sorokiniana* isolates revealed that few haplotypes were population-specific and unique, while others were evolutionarily distinctive (H_1) and exhibited significant levels of gene flow among populations. The Asian haplotypes H_2, H_4, H_6, H_7, H_12, H_15, H_16, H_17, H_18, H_19, H_20, H_21, H_22, H_23, H_25, H_26, and H_39 appeared to have evolved from the predominant haplotype H_1 (Figure 1). The haplotypes H_1, H_3, H_4, H_27, H_28, H_29, H_30, H_31, and H_40 were from barley isolates of India, Morocco, Syria, Turkey, Japan, China, and Syria origins, respectively (Supplementary Table S2). We found that Indian isolates were diverse and spread in the network in 16 haplotypes, i.e., H_1, H_2, H_5, H_9, H_10, H_11, H_12, H_13, H_14, H_29, H_30, H_33, H_34, H_35, H_36, and H_37. A star-like structure of the median joining network discovered in this study also offers evidence to support the presumption of divergence from neutrality for constant or uniform population size (Fu’s *Fs* and Tajima’s *D*) owing to spot blotch fungal population expansion. *B. sorokiniana* host plant introduction, domestication, and continuing cultivation may therefore be a plausible explanation for population growth in different wheat-producing zones. This type of fungal population movement has been observed across several fungal infections [43,44,45].

The indices were non-significantly positive in the Indian group, showing divergent demographic histories among the analysed regions. Tajimas’*D* was negative for most of the groups but statistically positive, and Fu’s *Fs* were negative for only Syria but statistically significant, indicating that the *B. sorokiniana* populations did not match a simple model of neutrality and rejected the null hypothesis of constant population size. These findings postulated that the divergence from neutrality for constant population size (Tajima’s *D* and Fu’s *Fs*) was caused by the recent population diversification. The introduction of *B. sorokiniana* might be the cause of the species’ recent population expansion, as *Bipolaris* now exhibits pathogenic specialization on a variety of wild and cultivated host plants. This type of range expansion has also been reported in many fungal and insect species [46,47]. The dating analysis shows that the species *B. Sorokiniana* originated from the Middle Miocene era (~19 Mya). The Miocene era had the longest and most humid environments in geological history. According to paleogeographic analysis, the western part of Central Asia transitioned from a humid climate to a semi-arid climate [48,49]. This climatic change is thought to have accelerated speciation due to habitat alternations caused by progressive acidification and the quaternary glacial–interglacial cycles. As a result, the climatic change results in contraction and speciation. Divergence times estimated for the primary genetic breaks were placed within the Plio/Pleistocene periods (~2 Mya) (Figure 4), a period in which the average temperature of the earth began to decrease rapidly and for which extensive records document changes in forest distribution associated with climatic cycles. The first break among the groups occurred in the Asian lineages of *B. sorokiniana* (the oldest ones). This study also showed that the divergence of *B. sorokiniana* in Asian countries occurred 0.47 million years ago. It was discovered that divergence between Indian isolates and American isolates began 0.35 Mya, whereas it was 0.05 Mya between African isolates. In conclusion, the presence of one haplotype at a very high frequency and low genetic differentiation in *B. sorokiniana* suggests that the durability of resistance in host plants against pathogens could be improved by the exchange of elite resistance lines from different countries. Based on the current distribution of species, divergence time analysis suggests that haplogroups began at the Plio/Pleistocene boundary.

## Figures and Tables

**Table 1:** Analysis of genetic diversity and neutrality test of *B. sorokiniana* representing different countries.

Population	N	Hd	π	S	K	Fu’ *Fs*	Tajima’s D	R^2^
India	161	0.74 ± 0.028	0.34 ± 0.01	273	96.5	44.4	0.278	0.175
Japan	11	0.76 ± 0.08	0.45 ± 0.06	208	101.78	22.85	−0.05	0.167
Syria	4	1 ± 0.177	0.009 ± 0.002	1	0.66	−0.361	0.389	0.182
USA	4	1 ± 0.17	0.28 ± 0.152	128	64	2.988	−0.88 ***	0.43
Argentina	6	1 ± 0.096	0.5 ± 0.107	202	114.5	2.78	−0.26	0.282
Jordan	4	0.5 ± 0.265	0.01 ± 0.006	2	1	4.419	−0.84	0.433
Egypt	3	1 ± 0.272	0.0896 ± 0.03	2	1.33	-	-	0.362
Turkey	8	0.57 ± 0.09	0.33 ± 0.05	291	166	27.43	2.64 ***	0.285
China	9	0.833 ± 0.127	0.240 ± 0.110	238	69.63	7.4	−1.796 *	0.215
Azerbaijan	10	0.64 ± 0.10	0.30 ± 0.0	306	163	27.17	2.43 **	0.264
Mexico	4	0.5 ± 0.265	0.0019 ± 0.	2	1	1.099	−0.709	0.433
Russia	5	1 ± 0.126	0.045 ± 0.012	51	27.2	0.874	−0.97	0.218
Kazakhstan	6	0.6 ± 0.12	0.34 ± 0.07	282	169	20.9	2.4	0.3
Morocco	4	0.8 ± 0.22	0.006 ± 0.002	6	3.16	0.811	−0.314	0.365
**Total**	**239**	**0.7 ± 0.027**	**0.33 ± 0.01**	**225**	**75.5**	**60.02**	**−0.105**	**0.3**

Note: N: Number of sequences; Hd = haplotype diversity; π = nucleotide diversity; S = variable sites; K = average number of nucleotide difference; Statistical significance: * *p* < 0.05; ** *p* < 0.01; *** *p* < 0.001; A negative Tajima’s D signifies an excess of low-frequency polymorphisms relative to expectation indicating population size expansion or positive selection; a negative Fu’ *Fs* value provides strong evidence for past population expansion, and rules out the possibility of background selection, and evolutionary forces that produce a pattern similar to population expansion.

**Table 2:** AMOVA of ITS, *GAPDH* and *TEF-1α* sequences for geographic and haplogroups of *B. sorokiniana*.

		F-Statistic		
Groups	Variation%	Փst	Փsc	Փct
Among populations	5.84681	0.05847	-	-
within populations	94.15319			
		Փst	Փsc	Փct
Among groups	7.44	0.182	0.233	−0.066
Among populations within groups	27.77			
Within population within groups	91.28			

## Data Availability

Not applicable.

## Supplementary Materials

The following supporting information can be downloaded at: https://www.mdpi.com/article/10.3390/genes13122206/s1, Table S1: List of sources of *B. sorokiniana isolates* used in this study; Table S2: Geographical origin, host, isolates, haplotypes information and their accession number of *B. sorokiniana* ITS, *GAPDH* and *TEF 1α* sequences used for genetic analysis.
