# Supplementary material for: Phylogeographic Diversity Analysis of Bipolaris sorokiniana (Sacc.) Shoemaker Causing Spot Blotch Disease in Wheat and Barley
Source: Genes (Basel). 2022 Nov 24;13(12):2206. doi: 10.3390/genes13122206 (PMC9778185; doi:10.3390/genes13122206)
Supplement: Supplementary file 1 [file genes-13-02206-s001.zip › genes-2005040-supplementary.pdf]

Table S1. List of source of *Bipolaris sorokiniana*isolates used in this study.

Sr no.	Populations locations	Isolates	Latitude (DMS)	Host plant	Gene Bank accession
1.	Faizabad, Uttar Pradesh	BBS -1	26° 46' 12" N	Barley	KT864928
2.	Varanasi, Uttar Pradesh	BBS-3	25° 19' 18.0624" N	Barley	KT864929
3.	Ludhiana, Punjab	BBS-4	30° 54' 3.474" N	Barley	KT864930
4.	Dharwad, Karnataka	BBS-5	16°.14' N	Barley	KT864931
5.	Dharwad, Karnataka	WBS-1	16°.14' N	Wheat	KT864932
6.	Gohana, Haryana	WBS-2	29° 8' 18.2652" N	Wheat	KT864933
7.	Hosangabad, Madhya Pradesh	WBS-3	22° 45' 12.852" N	Wheat	KT864934
8.	Indore, Madhya Pradesh	WBS-4	22° 43' 10.4448" N	Wheat	KT864935
9.	Meerut, Uttar Pradesh	WBS-6	28° 59' 4.7184" N	Wheat	KT884113
10.	Muzaffarnagar , Uttar Pradesh	WBS-7	29° 26' 0.042" N	Wheat	KT884114
11.	Pantnagar, Uttarakhand	WBS-8	29° 1' 15.7368" N	Wheat	KT884115
12.	Pune, Maharashtra	WBS-9	18° 31' 0.2136" N	Wheat	KT884116
13.	Pusa, Bihar	WBS-10	25° 59' 5.0496" N	Wheat	KT884117
14.	Rhotak, Haryana	WBS-11	28° 53' 40.092" N	Wheat	KT884118
15.	Saharanpur, Haryana	WBS-12	29° 57' 34.8984" N	Wheat	KT884119
16.	Karnal, Haryana	WBS-13	29° 41' 8.2644" N	Wheat	KT884120
17.	Kurukshetra, Haryana	WBS-14	29° 58' 10.2468" N	Wheat	KT884121
18.	Ludhiana, Punjab	WBS-15	30° 58' 44.1588" N	Wheat	KT884122
19.	Dharwad, Karnataka	WBS-16	15° 20' 49.6896" N	Wheat	KT884123
20.	Gurdaspur, Himachal Pradesh	WBS-17	32°2'30.99"N	Wheat	KT884124
21.	Shamli, Uttar Pradesh	WBS-18	29° 31' 0" N	Wheat	KT884125

Table S2. Geographical origin, host, isolates, haplotypes information and their accession number of *B. sorokiniana* ITS, *GAPDH* and *TEF 1α* sequences used for genetic analysis.

Isolates	Accession No.	Gene	Country	Host	Tissue type	Haplotypes
BHU RC 9	MH209068	ITS	India (North India)	wheat	Seed, leaves	H1
MDSN24-D-16	MH209067	ITS	India (North India)	wheat	Seed, leaves	H1
K0911	MH209066	ITS	India (North India)	wheat	Seed, leaves	H1
D6899	MH209065	ITS	India (North India)	wheat	Seed, leaves	H26
UBS2	MH209063	ITS	India (North India)	wheat	Seed, leaves	H1
RAJ3975	MH209058	ITS	India (North India)	wheat	Seed, leaves	H1
BHU RC-8	MH209057	ITS	India (North India)	wheat	Seed, leaves	H1
W	MH209056	ITS	India (North India)	wheat	Seed, leaves	H15
Barley 356	MH209055	ITS	India (North India)	wheat	Seed, leaves	H16
KO-5803	MH209054	ITS	India (North India)	wheat	Seed, leaves	H17
HI1461	MH209053	ITS	India (North India)	wheat	Seed, leaves	H18
S-13	MH209052	ITS	India (North India)	wheat	Seed, leaves	H1
HP-1744	MH209051	ITS	India (North India)	wheat	Seed, leaves	H1
B-4-2-12	MH209050	ITS	India (North India)	wheat	Seed, leaves	H1
B-10-A	MH209049	ITS	India (North India)	wheat	Seed, leaves	H1
MP1261	MH209047	ITS	India (North India)	wheat	Seed, leaves	H1
D-6-1-6	MH209046	ITS	India (North India)	wheat	Seed, leaves	H1
HP1493	MH209045	ITS	India (North India)	wheat	Seed, leaves	H1
BHU RC-6	MH208996	ITS	India (North India)	wheat	Seed, leaves	H1
RAJ4252	MH209044	ITS	India (North India)	wheat	Seed, leaves	H19
T-1	MH209043	ITS	India (North India)	wheat	Seed, leaves	H20
DBW14	MH209008	ITS	India (North India)	wheat	Seed, leaves	H1
BHU RC10	MH209009	ITS	India (North India)	wheat	Seed, leaves	H21
HD2888	MH209041	ITS	India (North India)	wheat	Seed, leaves	H1
MDSN76-DB-16	MH209040	ITS	India (North India)	wheat	Seed, leaves	H1
RSP561	MH209039	ITS	India (North India)	wheat	Seed, leaves	H1
UBS1	MH209038	ITS	India (North India)	wheat	Seed, leaves	H1
94 LOK 67-19-2	MH209037	ITS	India (North India)	wheat	Seed, leaves	H21
HUW16	MH209036	ITS	India (North India)	wheat	Seed, leaves	H22
BHU RC-1	MH209035	ITS	India (North India)	wheat	Seed, leaves	H1
K1013-1	MH209034	ITS	India (North India)	wheat	Seed, leaves	H24
MP1266, WBI-B	MH209033	ITS	India (North India)	wheat	Seed, leaves	H1
BR3705	MH209032	ITS	India (North India)	wheat	Seed, leaves	H1
S-913-14	MH209031	ITS	India (North India)	wheat	Seed, leaves	H1
B7	MH209030	ITS	India (North India)	wheat	Seed, leaves	H1
HD2329	MH209029	ITS	India (North India)	wheat	Seed, leaves	H1
BHU-RC-4	MH197356	ITS	India (North India)	wheat	Seed, leaves	H1
CG80001	MH209028	ITS	India (North India)	wheat	Seed, leaves	H1
S-13-4-C25	MH209027	ITS	India (North India)	wheat	Seed, leaves	H1
70B-1002	MH209026	ITS	India (North India)	wheat	Seed, leaves	H1
DBW91	MH197355	ITS	India (North India)	wheat	Seed, leaves	H1
HD3091	MH209024	ITS	India (North India)	wheat	Seed, leaves	H1
SeedR2	MH209023	ITS	India (North India)	wheat	Seed, leaves	H1

S-13-3	MH209022	ITS	India (North India)	wheat	Seed, leaves	H25
BBS -1	KT864928	ITS	India (North India)	Barley	Seed	H1
BBS-3	KT864929	ITS	India (North India)	Barley	Seed	H1
BBS-4	KT864930	ITS	India (North India)	Barley	Seed	H1
BBS-5	KT864931	ITS	India (North India)	Barley	Seed	H1
WBS-1	KT864932	ITS	India (North India)	wheat	Seed	H1
WBS-2	KT864933	ITS	India (North India)	wheat	Seed	H1
WBS-3	KT864934	ITS	India (North India)	wheat	Seed	H1
WBS-4	KT864935	ITS	India (North India)	wheat	Seed	H1
WBS-6	KT884113	ITS	India (North India)	wheat	Seed	H1
WBS-7	KT884114	ITS	India (North India)	wheat	Seed	H1
WBS-8	KT884115	ITS	India (North India)	wheat	Seed	H1
WBS-9	KT884116	ITS	India (North India)	wheat	Seed	H1
WBS-10	KT884117	ITS	India (North India)	wheat	Seed	H1
WBS-11	KT884118	ITS	India (North India)	wheat	Seed	H5
WBS-12	KT884119	ITS	India (North India)	wheat	Seed	H1
WBS-13	KT884120	ITS	India (North India)	wheat	Seed	H1
WBS-14	KT884121	ITS	India (North India)	wheat	Seed	H1
WBS-15	KT884122	ITS	India (North India)	wheat	Seed	H1
WBS-16	KT884123	ITS	India (North India)	wheat	Seed	H1
WBS-17	KT884124	ITS	India (North India)	wheat	Seed	H1
WBS-18	KT884125	ITS	India (North India)	wheat	Seed	H1
WLB-18-20	MK809545	ITS	India (Central India)	wheat	NA	H9
WLB-18-7	MK809556	ITS	India (Central India)	wheat	NA	H2
Isolate L	KJ562717	ITS	India (Central India)	wheat	NA	H1
Isolate D2	KJ562716	ITS	India (Central India)	wheat	NA	H1
Isolate J	KJ562715	ITS	India (Central India)	wheat	NA	H1
HD3069	KJ562714	ITS	India (Central India)	wheat	NA	H1
WLB-18-5	MK809546	ITS	India (Central India)	wheat	NA	H2
WLB-18-8	MK809555	ITS	India (Central India)	wheat	NA	H2
BS69	HM195257	ITS	India (Central India)	wheat	NA	H1
Isolate A	KJ562718	ITS	India (South India)	wheat	NA	H1
Dharwad	KJ562719	ITS	India (South India)	wheat	NA	H1
Isolate SHZ-BH2	MK640670	ITS	India (South India)	wheat	NA	H1
BS72	HM195258	ITS	India (South India)	wheat	NA	H1
BS75	HM195259	ITS	India (South India)	wheat	NA	H1
BS77	HM195260	ITS	India (South India)	wheat	NA	H1
BS79	HM195261	ITS	India (South India)	wheat	NA	H1
BS92	HM195262	ITS	India (South India)	wheat	NA	H1
SHZ-BS-3	MK777970	ITS	India (South India)	wheat	NA	H1
WH.PBW.1P.04	KM066949	ITS	India (West India)	wheat	NA	H1
WLB-18-13	MK809551	ITS	India (West India)	wheat	NA	H2
WB3	MT804344	ITS	India (West India)	wheat	NA	H1
WB4	MT804345	ITS	India (West India)	wheat	NA	H1
WB6	MT804347	ITS	India (West India)	wheat	NA	H1
WB7	MT804348	ITS	India (West India)	wheat	NA	H1

WLB-17-2	MK809564	ITS	India (West India)	wheat	NA	H10
WLB-17-1	MK809563	ITS	India (West India)	wheat	NA	H11
BS41	HM195251	ITS	India (West India)	wheat	NA	H1
BS42	HM195252	ITS	India (West India)	wheat	NA	H1
WLB-18-31	MK809558	ITS	India (West India)	wheat	NA	H12
WLB-18-9	MK809561	ITS	India (West India)	wheat	NA	H13
WLB-17-47	MN535889	ITS	India (West India)	wheat	NA	H14
WLB-18-22	MK809565	ITS	India (West India)	wheat	NA	H2
WLB-18-33	MK809566	ITS	India (West India)	wheat	NA	H2
BS-47	DQ367884	ITS	India (West India)	wheat	NA	H1
B131-2	LC543651	ITS	Japan	Barley	Spike	H1
BMup2	LC543650	ITS	Japan	Barley	Spike	H1
BM2	LC543649	ITS	Japan	Barley	Spike	H1
BO2	LC543648	ITS	Japan	Barley	Spike	H1
J1	MT035843.1	ITS	China	Wheat, Barley	NA	H1
J10	MT035842.1	ITS	China	Wheat, Barley	NA	H1
GN1	MN736413.1	ITS	China	Wheat, Barley	NA	H1
YT1-2-2	KJ026145.1	ITS	China	Wheat, Barley	NA	H1
WK1-3-6	KJ026144.1	ITS	China	Wheat, Barley	NA	H1
RBC3	KJ026140.1	ITS	China	Wheat, Barley	NA	H8
isolate 83	MN534829.1	ITS	Jordan	Wheat	NA	H1
isolate 49	MN534803.1	ITS	Jordan	Wheat	NA	H1
isolate 112	MN534844.1	ITS	Jordan	Wheat	NA	H6
isolate 89	MN534835.1	ITS	Jordan	Wheat	NA	H1
isolate 54_F	MN444784.1	ITS	Morocco	Barley	NA	H1
isolate C404-11	MN313300.1	ITS	Canada	Wheat	NA	H1
isolate C109-13	MN313291.1	ITS	Canada	Wheat	NA	H1
isolate Mm51	MK156316.1	ITS	Iran	Wheat	NA	H1
isolate Mm50	MK156315.1	ITS	Iran	Wheat	NA	H1
isolate Mm41	MK156311.1	ITS	Iran	Wheat	NA	H1
isolate 72	MG654434.1	ITS	Azerbaijan	Wheat	root	H1
isolate 71	MG654433.1	ITS	Azerbaijan	Wheat	root	H1
isolate 66	MG654432.1	ITS	Azerbaijan	Wheat	root	H1
strain 69	MG661715.1	ITS	Azerbaijan	Wheat	root	H1
strain_69	MK022351.1	ITS	Azerbaijan	Wheat	root	H1
strain_68	MK022350.1	ITS	Azerbaijan	Wheat	root	H1
strainAUMC14512	MT974154.1	ITS	Egypt	Wheat	Stored grains	H1
isolate Bs12	MW246488.1	ITS	Egypt	Wheat	Stored grains	H1
isolate Bs13	MW246567.1	ITS	Egypt	Wheat	Stored grains	H7
isolate TPQ5	KX137838.1	ITS	Mexico	Wheat	NA	H1
isolate TPQ4	KX137837.1	ITS	Mexico	Wheat	NA	H1

isolate TPQ3	KX137836.1	ITS	Mexico	Wheat	NA	H1
isolate TPQ2	KX137835.1	ITS	Mexico	Wheat	NA	H1
isolate 10	KC616341.1	ITS	Syria	Barley	NA	H3
isolate 49	KC616339.1	ITS	Syria	Barley	NA	H3
isolate C9	KC616337.1	ITS	Syria	Barley	NA	H4
isolate 92	KC616335.1	ITS	Syria	Barley	NA	H4
isolate CS1	JX145649.1	ITS	USA	Wheat	NA	H27
ATCC 201653	JQ070091.1	ITS	USA	Wheat	NA	H1
ATCC 201652	JQ070090.1	ITS	USA	Wheat	NA	H1
TR-Cs-8	MT271247.1	ITS	Turkey	Wheat,barley	Soil	H1
TR-Cs-10	MT271249.1	ITS	Turkey	Wheat, Barley	Soil	H1
TR-Cs-6	MT271245.1	ITS	Turkey	Wheat, Barley	Soil	H1
TR-Cs-4	MT271243.1	ITS	Turkey	Wheat, Barley	Soil	H1
AB-F 67	KX622098.1	ITS	Russia	Seed of cereals	NA	H1
AB-F 83	KX622114.1	ITS	Russia	Seed of cereals	NA	H1
AB-F 81	KX622112.1	ITS	Russia	Seed of cereals	NA	H1
AB-F 79	KX622110.1	ITS	Russia	Seed of cereals	NA	H1
AB-F 61	KX622092.1	ITS	Russia	Seed of cereals	NA	H1
strain 98026P	KF765405.1	ITS	Brazil	Wheat	NA	H1
strain 98023B	KF765406.1	ITS	Brazil	Wheat	NA	H1
strain 98023P	KF765407.1	ITS	Brazil	Wheat	NA	H1
strain 98026C	KF765408.1	ITS	Brazil	Wheat	NA	H1
BHU RC 9	MH515071	*TEF-α1*	India (North India)	wheat	NA	H29
MDSN24-D-16	MH515070	*TEF-α1*	India (North India)	wheat	NA	H29
K0911	MH515069	*TEF-α1*	India (North India)	wheat	NA	H29
D6899	MH515068	*TEF-α1*	India (North India)	wheat	NA	H39
UBS2	MH515067	*TEF-α1*	India (North India)	wheat	NA	H29
RAJ3975	MH515066	*TEF-α1*	India (North India)	wheat	NA	H29
BHU RC-8	MH515065	*TEF-α1*	India (North India)	wheat	NA	H29
W	MH515064	*TEF-α1*	India (North India)	wheat	NA	H29
Barley 356	MH515063	*TEF-α1*	India (North India)	wheat	NA	H29
KO-5803	MH515062	*TEF-α1*	India (North India)	wheat	NA	H29
HI1461	MH515061	*TEF-α1*	India (North India)	wheat	NA	H29
S-13	MH515060	*TEF-α1*	India (North India)	wheat	NA	H29
HP-1744	MH515059	*TEF-α1*	India (North India)	wheat	NA	H29
B-4-2-12	MH515058	*TEF-α1*	India (North India)	wheat	NA	H29
B-10-A	MH515057	*TEF-α1*	India (North India)	wheat	NA	H29
MP1261	MH515056	*TEF-α1*	India (North India)	wheat	NA	H29
D-6-1-6	MH515055	*TEF-α1*	India (North India)	wheat	NA	H29

HP1493	MH515054	*TEF-α1*	India (North India)	wheat	NA	H29
BHU RC-6	MH515005	*TEF-α1*	India (North India)	wheat	NA	H29
RAJ4252	MH515052	*TEF-α1*	India (North India)	wheat	NA	H29
T-1	MH515051	*TEF-α1*	India (North India)	wheat	NA	H29
DBW14	MH515017	*TEF-α1*	India (North India)	wheat	NA	H29
HD2888	MH515049	*TEF-α1*	India (North India)	wheat	NA	H29
MDSN76-DB-16	MH515048	*TEF-α1*	India (North India)	wheat	NA	H29
RSP561	MH515047	*TEF-α1*	India (North India)	wheat	NA	H29
UBS1	MH515046	*TEF-α1*	India (North India)	wheat	NA	H29
94 LOK 67-19-2	MH515045	*TEF-α1*	India (North India)	wheat	NA	H29
HUW16	MH515044	*TEF-α1*	India (North India)	wheat	NA	H29
BHU RC-1	MH515043	*TEF-α1*	India (North India)	wheat	NA	H29
K1013-1	MH515042	*TEF-α1*	India (North India)	wheat	NA	H29
MP1266, WBI-B	MH515041	*TEF-α1*	India (North India)	wheat	NA	H29
BR3705	MH515040	*TEF-α1*	India (North India)	wheat	NA	H29
S-913-14	MH515039	*TEF-α1*	India (North India)	wheat	NA	H29
B7	MH515038	*TEF-α1*	India (North India)	wheat	NA	H29
HD2329	MH515037	*TEF-α1*	India (North India)	wheat	NA	H29
BHU-RC-4	MH515036	*TEF-α1*	India (North India)	wheat	NA	H29
CG80001	MH515035	*TEF-α1*	India (North India)	wheat	NA	H29
S-13-4-C25	MH515034	*TEF-α1*	India (North India)	wheat	NA	H29
70B-1002	MH515033	*TEF-α1*	India (North India)	wheat	NA	H29
DBW91	MH515032	*TEF-α1*	India (North India)	wheat	NA	H29
HD3091	MH515031	*TEF-α1*	India (North India)	wheat	NA	H29
SeedR2	MH515030	*TEF-α1*	India (North India)	wheat	NA	H29
S-13-3	MH515029	*TEF-α1*	India (North India)	wheat	NA	H29
B131-2	LC546960	*TEF-α1*	Japan	Barley	NA	H31
BMup2	LC546959	*TEF-α1*	Japan	Barley	NA	H31
BM2	LC546958	*TEF-α1*	Japan	Barley	NA	H31
BO2	LC546957	*TEF-α1*	Japan	Barley	NA	H31
WLB-18-43	MW085804	*TEF-α1*	India (North India)	wheat	NA	H33
WLB-18-31	MW085803	*TEF-α1*	India (North India)	wheat	NA	H34
WLB-18-24	MW085802	*TEF-α1*	India (North India)	wheat	NA	H33
WLB-18-20	MW085801	*TEF-α1*	India (North India)	wheat	NA	H33
WLB-18-11	MW085800	*TEF-α1*	India (North India)	wheat	NA	H33
WLB-18-10	MW085799	*TEF-α1*	India (North India)	wheat	NA	H35
WLB-18-9	MW085798	*TEF-α1*	India (North India)	wheat	NA	H33
WLB-18-7	MW085797	*TEF-α1*	India (North India)	wheat	NA	H33
WLB-18-6	MW085796	*TEF-α1*	India (North India)	wheat	NA	H36
WLB-18-1	MW085795	*TEF-α1*	India (North India)	wheat	NA	H30
WLB-17-55	MW085794	*TEF-α1*	India (North India)	wheat	NA	H37
WLB-17-47	MW085793	*TEF-α1*	India (North India)	wheat	NA	H30
WLB-17-13	MW085792	*TEF-α1*	India (North India)	wheat	NA	H33
WLB-17-2	MW085791	*TEF-α1*	India (North India)	wheat	NA	H30
WLB-17-1	MW085790	*TEF-α1*	India (North India)	wheat	NA	H30
WLB-10-6	MW085789	*TEF-α1*	India (North India)	wheat	NA	H38

WLB-10-3	MW085788	*TEF-α1*	India (North India)	wheat	NA	H33
MAFF 238877	KM093765.1	*TEF-α1*	Japan	Barley	NA	H30
M1	KM243317.1	*TEF-α1*	China	wheat	NA	H32
CPC 28832	MF490855.1	*TEF-α1*	Thailand	wheat	NA	H29
B7	MZ327275	*TEF-α1*	Argentina	Barley	seed	H40
B19	MW802821	*TEF-α1*	Argentina	Barley	seed	H30
B13	MZ188975	*TEF-α1*	Argentina	Barley	seed	H29
K1012	MH279672	*TEF-α1*	India (North India)	wheat	Leaves	H29
B15	KJ939504	*TEF-α1*	India (North India)	wheat	Na	H30
B11	KJ939501	*TEF-α1*	India (North India)	wheat	Na	H30
CS1	JX145648	*GAPDH*	USA	wheat	Na	H27
Azerbaijan 69	MH844821	*GAPDH*	Azerbaijan	wheat	Na	H27
Azerbaijan 68	MH844820	*GAPDH*	Azerbaijan	wheat	Na	H27
Azerbaijan 60	MH844819	*GAPDH*	Azerbaijan	wheat	Na	H27
Azerbaijan 15	MH844818	*GAPDH*	Azerbaijan	wheat	Na	H27
strain 54-2	MK024323	*GAPDH*	Turkey	wheat	Na	H27
strain 47	MK024322	*GAPDH*	Turkey	wheat	Na	H27
strain 30-2	MK024321	*GAPDH*	Turkey	wheat	Na	H27
strain 23-3	MK024320	*GAPDH*	Turkey	wheat	Na	H27
M1	KR527151	*GAPDH*	China	wheat	Na	H27
MAFF 238877	KM034824	*GAPDH*	Japan	Barley	Na	H27
MAFF 235500	KM034823	*GAPDH*	Japan	Barley	Na	H27
Isolate 03	MT262924	*GAPDH*	Kazakhstan	triticale	Na	H27
isolate 02	MT262923	*GAPDH*	Kazakhstan	triticale	Na	H27
isolate 01	MT262922	*GAPDH*	Kazakhstan	triticale	Na	H27
B7	MZ156796	*GAPDH*	Argentina	Barley	Seed	H27
B_19	MW980141	*GAPDH*	Argentina	Barley	Seed	H28
B13	MZ327274	*GAPDH*	Argentina	Barley	Seed	H27
